# T-cell exhaustion-related genes in Graves’ disease: a comprehensive genome mapping analysis

**DOI:** 10.3389/fendo.2024.1364782

**Published:** 2024-08-22

**Authors:** Zhengrong Jiang, Huiyao Cai, Yizhao Lin, Ruhai Lin, Lijun Chen, Huibin Huang

**Affiliations:** ^1^ Department of Endocrinology, Second Affiliated Hospital of Fujian Medical University, Quanzhou, Fujian, China; ^2^ Department of Internal Medicine, Gutian County Hospital of Ningde City, Ningde, Fujian, China

**Keywords:** Graves’ disease, T-cell exhaustion, weighted gene co-expression network analysis, *CBL*, enlargement of thyroid gland

## Abstract

**Background:**

T-cell exhaustion (Tex) can be beneficial in autoimmune diseases, but its role in Graves’ disease (GD), an autoimmune disorder of the thyroid, remains unknown. This study investigated Tex-related gene expression in GD patients to discern the potential contributions of these genes to GD pathogenesis and immune regulation.

**Methods:**

Through gene landscape analysis, a protein–protein interaction network of 40 Tex-related genes was constructed. mRNA expression levels were compared between GD patients and healthy control (HCs). Unsupervised clustering categorized GD cases into subtypes, revealing distinctions in gene expression, immune cell infiltration, and immune responses. Weighted gene co-expression network analysis and differential gene expression profiling identified potential therapeutic targets. RT-qPCR validation of candidate gene expression was performed using blood samples from 112 GD patients. Correlations between Tex-related gene expression and clinical indicators were analyzed.

**Results:**

Extensive Tex-related gene interactions were observed, with six genes displaying aberrant expression in GD patients. This was associated with atypical immune cell infiltration and regulation. Cluster analysis delineated two GD subtypes, revealing notable variations in gene expression and immune responses. Screening efforts identified diverse drug candidates for GD treatment. The Tex-related gene *CBL* was identified for further validation and showed reduced mRNA expression in GD patients, especially in cases of relapse. *CBL* mRNA expression was significantly lower in patients with moderate-to-severe thyroid enlargement than in those without such enlargement. Additionally, *CBL* mRNA expression was negatively correlated with the disease-specific indicator thyrotropin receptor antibodies.

**Conclusion:**

Tex-related genes modulate GD pathogenesis, and their grouping aids subtype differentiation and exploration of therapeutic targets. *CBL* represents a potential marker for GD recurrence.

## Introduction

Graves’ disease (GD) is a prevalent autoimmune thyroid disorder that affects an estimated 0.5–2% of the global population (1). GD commonly manifests as symptomatic thyrotoxicosis, dysregulated hormone levels, and the presence of thyrotropin receptor antibodies (TRAb), conditions that can lead to life-threatening hyperthyroid crisis in some cases, leading to considerable morbidity and mortality (2). A thorough understanding of the etiology and pathogenesis of GD is necessary for the development of precise diagnostic methods and effective therapeutic interventions. Central to GD pathology is discernible derangement of T-cell functionality, which prompts a cascade of events that lead to the breakdown of immune tolerance, aberrant immunoregulation, heightened B-cell reactivity, and ultimately injury to the thyroid and associated tissues (3). However, the nuanced mechanisms responsible for the alterations in T-cell function observed in GD remain incompletely understood.

T lymphocytes, commonly referred to as T cells, assume a central role as effectors in the immune system and are mobilized in response to antigenic stimuli. This orchestrated response entails rapid T-cell proliferation and differentiation, culminating in the development of both effector T cells and memory T cells—a process indispensable for the preservation of immune homeostasis. Dysregulation of T-cell function is intricately associated with the onset of autoimmune pathologies (4). Specifically, T-cell exhaustion (Tex) represents a consequential deviation in T-cell differentiation, evoked by prolonged exposure to antigenic stimulation and/or inflammation (5). Hallmarks of Tex include progressive functional decline, sustained elevation of inhibitory receptors, alterations in transcription factor expression and utilization, and metabolic perturbations. Importantly, exhausted T cells lack the antigen-independent homeostatic proliferative capacity exhibited by memory T cells (6). The well-established connection of Tex with viral infections and compromised anti-tumor responses highlights its pivotal role. Within the tumor microenvironment, Tex may contribute to a waning anti-tumor immune response, fostering immune tolerance. Conversely, in autoimmune diseases, Tex may be beneficial, as depleted T cells modulate autoimmune responses through immunoregulatory mechanisms, thereby contributing to the restoration of immune homeostasis (7). Existing research underscores the correlation between the transcriptional profile of Tex and the therapeutic efficacy and prognosis across diverse autoimmune diseases, including systemic lupus erythematosus (SLE), type 1 diabetes mellitus, antineutrophil cytoplasmic antibody-associated vasculitis disease, rheumatoid arthritis (RA), and idiopathic pulmonary fibrosis (8). Although Tex has been studied in many autoimmune diseases, little evidence for its role in GD can be found in the current literature. An in-depth exploration of Tex within the context of GD would provide important insight into the precise mechanisms that underlie abnormal T-cell function in this specific autoimmune thyroid disorder.

In the present study, we first delineated molecular subtypes of GD by scrutinizing the expression profiles of Tex-related genes in the peripheral blood mononuclear cells (PBMCs) of GD patients. Through a meticulous analysis of Tex-related gene expression, we elucidated distinctive immune characteristics across these identified subtypes. For these subtypes, we performed co-expression analyses to identify key molecules that could serve as targets for potential molecularly-targeted drugs. This pioneering approach not only sheds light on the intricate immune landscape in GD but also introduces a novel research avenue for the prospective diagnosis and treatment of this autoimmune thyroid disorder.

## Materials and methods

### Data collection and pre-processing

For use in this study, the GSE71956 dataset was retrieved from the NCBI’s GEO database (https://www.ncbi.nlm.nih.gov/geo/). This microarray dataset, focusing on *Homo sapiens*, includes gene expression data collected from the PBMCs of 31 GD patients and 18 healthy controls (HCs), for a total of 49 samples (9, 10).

Data processing involved translating probes into symbols based on platform-specific correspondences. Probes corresponding to multiple genes were carefully removed, and those converging on the same symbol were consolidated into a median value. Subsequently, 40 Tex-related genes were identified (11). Gene sets from the GOBP and KEGG pathways, obtained from the MSigDb database (https://www.gsea-msigdb.org/gsea/msigdb/, version msigdb_v2023.1.Hs_GMTs), were included in further analysis using the ssGSEA algorithm to quantify pathway activity scores. Concurrently, we retrieved a set of 17 immune response genes from the Immport database (https://www.immport.org/shared/genelists). Utilizing the ssGSEA algorithm, immune response scores were computed for each sample in the GSE71956 dataset. The study also included 17 human leukocyte antigen (HLA) genes (12), and correlation analyses were conducted for 13 relevant genes. The four genes not included in the latter analyses were *HLA-J* and *HLA-DRB6*, which were absent from the GSE71956 dataset, and *HLA-A* and *HLA-B*, which were previously identified as key drivers in Tex.

### Protein-protein interaction network construction

Interactions among Tex-related genes were systematically explored using the STRING protein interactions database (https://www.string-db.org/). These interactions formed the basis for constructing PPI networks.

### Identification of differentially expressed Tex-related genes in GD patients

The Wilcoxon rank-sum test, with a stringent significance threshold set at *P*<0.05, was applied to identify differential expression of Tex-related genes between two delineated groups.

### Classification of GD subtypes using expression profiles of Tex-related genes

Unsupervised cluster analysis, utilizing the ConsensusClusterPlus package, identified different disease subtypes based on the expression profiles of Tex-related genes. The clustering method employed Euclidean distance, with km clustering and 1000 replications to ensure stability. Differences in pathway activity, immune response score, HLA gene expression, immune cell infiltration score, and T-cell depletion-related gene expression were examined between subtypes.

### Identification of key module genes in GD subtypes

To construct scale-free co-expression gene networks by weighted gene co-expression network analysis (WGCNA), stringent criteria included setting scale-free R^2^ to 0.8 and a minimum threshold of 30 genes within a module. Subsequently, correlation analyses evaluated the relationship between modules and sample phenotypic features, using Pearson’s correlation coefficients for Module Eigenvectors (MEs), signifying the most emblematic genes within each module. Core genes, integral to different subtypes, were identified through rigorous screening based on gene significance (GS) ≥0.6 and module membership (MM) ≥0.8.

### Differential gene expression analysis across GD subtypes

The R-package limma identified differentially expressed genes (DEGs) within diverse subtype samples, employing stringent criteria of |log2FC| ≥1 and adj. *P*<0.05.

### Functional enrichment analysis across GD subtypes

Functional annotation of DEGs between subtypes was completed using the R package clusterProfiler, with parameters including pvalueCutoff = 0.05 and pAdjustMethod = “BH.” This facilitated the annotation of potential functions of genes that diverged between subtypes, predicting their molecular functions.

### Identification of subtype hub genes and exploration of therapeutics via PPIs

WGCNA was performed to identify core genes for each subtype. Then PPI networks were constructed based on core DEGs. Subtype hub genes were selected based on those with the top 5 connectivity, and interactions between these genes and drugs were investigated using the DGIdb database (https://www.dgidb.org/, v4.2.0). Sankey diagrams were prepared to provide concise and informative visualization of the interactions between drugs and genes.

### Participant inclusion and blood sample collection

Peripheral blood samples were systematically collected from 112 GD patients and 47 HCs at the Second Hospital of Fujian Medical University between June 2023 and November 2023. The diagnostic criteria for GD encompassed clinical manifestations of thyrotoxicity, elevated serum thyrotropin receptor antibodies (TRAb), reduced serum thyrotropin (TSH), and other pertinent indicators (13). Individuals in the HC group had normal thyroid function and thyroid autoantibody levels, and individuals were excluded if they had a chronic inflammatory or autoimmune disorder, a prior thyroid condition or surgery, an infectious disease, or a malignant tumor, or if they were pregnant or breastfeeding.

Within the GD patient cohort, 70 cases were primary, signifying the patients had not received previous treatment for thyroid or ocular conditions. Based on the degree of thyroid enlargement (confirmed by palpation and ultrasound), these 70 patients were categorized into two groups: those without thyroid enlargement or with Grade I enlargement were included in the non-severe thyroid enlargement GD group (NSTEGD), while those with Grade II-III enlargement were included in the severe thyroid enlargement GD group (STEGD). In contrast, 42 cases were recurrent, marked by the recurrence of symptoms and serological changes meeting the diagnostic criteria for GD within 2 years of discontinuing prescribed medication, as directed by the treating physician (14).

Ethical approval was obtained from the Ethics Committee of the Second Hospital of Fujian Medical University (Ethics No. 2023-488). The general information of the study participants is presented in **Supplementary Table S1**. PBMCs were isolated through concentration gradient centrifugation and cryopreserved in serum-free cryopreservative for backup.

### RNA extraction and reverse transcription quantitative polymerase chain reaction

Total RNA was isolated from PBMCs using TRIzol (Thermo Fisher Scientific, Waltham, MA, USA) for reverse transcription. The resulting RNA was transcribed into cDNA using the PrimeScript RT Master Mix kit (Takara, Dalian, China), and then cDNA amplification was achieved using the TB Green Premix Ex Taq II kit (Takara). Primers targeting the CBL gene and *GAPDH* were synthesized by Shanghai Sangon (Shanghai, China). The experiment was repeated three times. The specific primer sequences were:

CBL-forward:5’-ACTGCTTCTAAGGCTGCTTCTGG-3’,R:5’-GGCGGTGGTGGTGGAAGATC-3’.GAPDH-forward:5’-TGCCACTCCTCCACCTTTG-3’,R:5’-CGAACCACCCTGTTGCTGT-3’.

### Statistical analysis

Bioinformatics analyses were conducted using R version 4.1.2. Test for significance included the Wilcoxon rank-sum test for two-group comparisons and the Kruskal–Wallis tests for multiple-group comparisons. The chromosomal distribution of Tex-related genes across 23 chromosome pairs was visualized using the R package RCircos.

Target gene validation was performed using IBM SPSS Statistics 23.0 and GraphPad Prism 8.0, employing the 2^-ΔCt^ method for relative gene expression in clinical samples. Normal data distribution was confirmed with the Shapiro–Wilk test, and data variance was assessed with the F-test. Normally distributed data were expressed as mean ± standard deviation, with between-group comparisons performed using the Student’s test or corrected t-test (Welch’s method). Non-normally distributed data were presented as median and quartiles, and between-group comparisons were performed used the Mann–Whitney test.

Correlations between the candidate gene *CBL* and FT3, FT4, and TSH levels were examined by Pearson’s correlation analysis, while the correlation between the candidate gene *CBL* and TRAb level was investigated by Spearman’s rank correlation analysis.

## Results

### Tex-related gene landscape in GD patients

**Figure 1:**
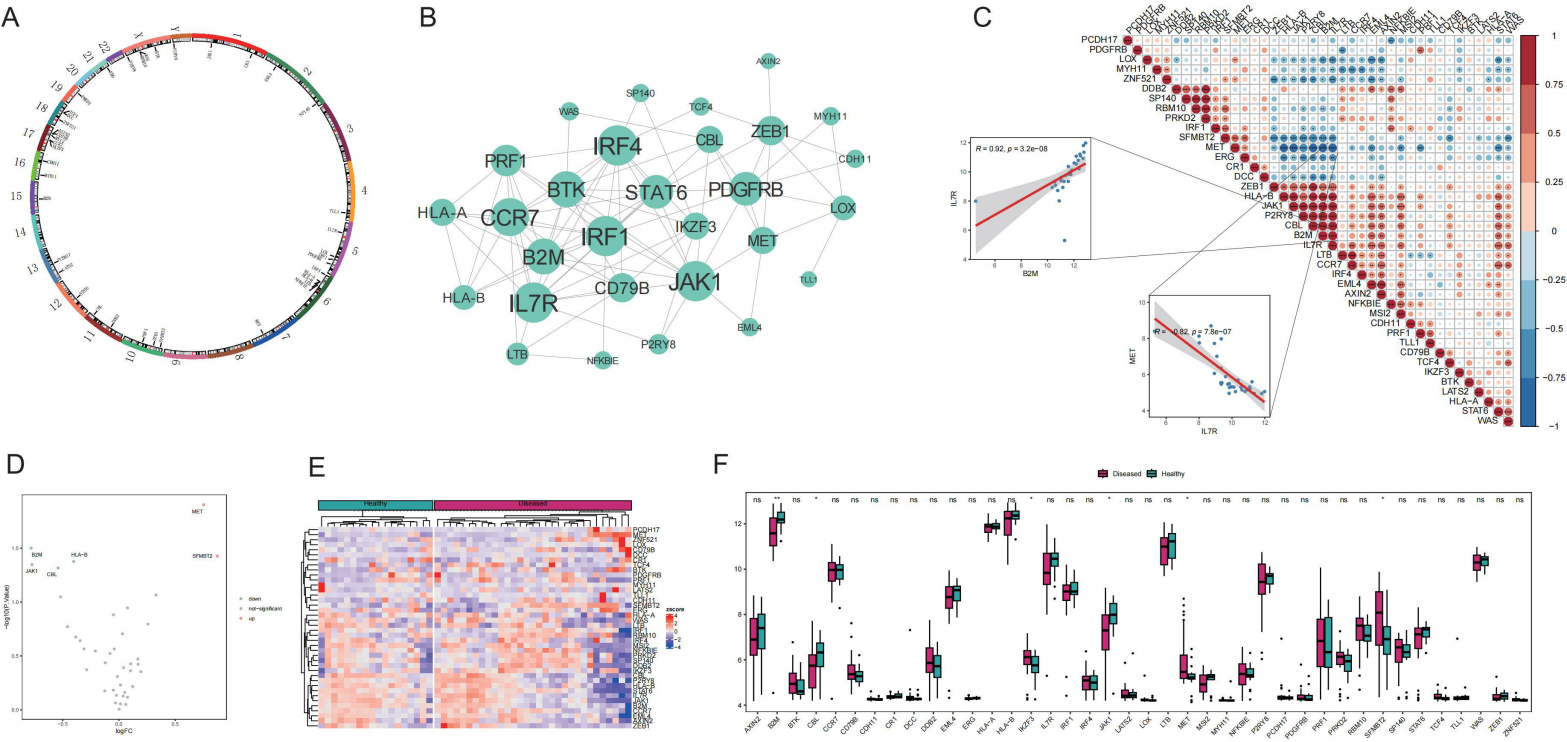
Expression of T-cell exhaustion (Tex)-related genes in the Graves’ disease (GD) genetic landscape. A: Chromosomal distribution of Tex-related genes. B: Protein–protein interaction (PPI) network of Tex-related genes. C: Heat map depicting expression correlation of Tex-related genes in disease samples. D: Volcano map illustrating differences in Tex-related gene expression between the healthy control (HC) and GD groups. E: Heat map displaying the expression levels of Tex-related genes in the HC and GD groups. F: Expression distribution map of differentially expressed Tex-related genes. ns (*p*>0.05), *P<0.05, **P<0.01.

This study began with a comprehensive analysis of 40 Tex-related genes, and the results revealed an even distribution across chromosomes, indicating genome-wide dispersion (**Figure 1A**). Subsequent PPI network analysis highlighted extensive interactions, emphasizing notable connectivity among genes such as *IL7R*, *CCR7*, *JAK1*, and *CBL* (**Figure 1B**), suggesting their potential importance in Tex-related gene interactions. Spearman’s correlation analysis demonstrated significant correlations in gene expression. The most pronounced was a positive correlation between *IL7R* and *B2M* expression, and a negative correlation between *IL7R* and *MET* exhibited the largest absolute value (**Figure 1C**), implying shared functional roles for these genes.

Comparison of the mRNA expression levels of Tex-related genes between healthy and GD samples revealed upregulation of two genes (*MET* and *SFMBT2*) and downregulation of four genes (*JAK1*, *CBL*, *B2M*, and *HLA-B*) in GD (**Figures 1D-F**).

### Involvement of Tex-related genes in immune regulation of GD

**Figure 2:**
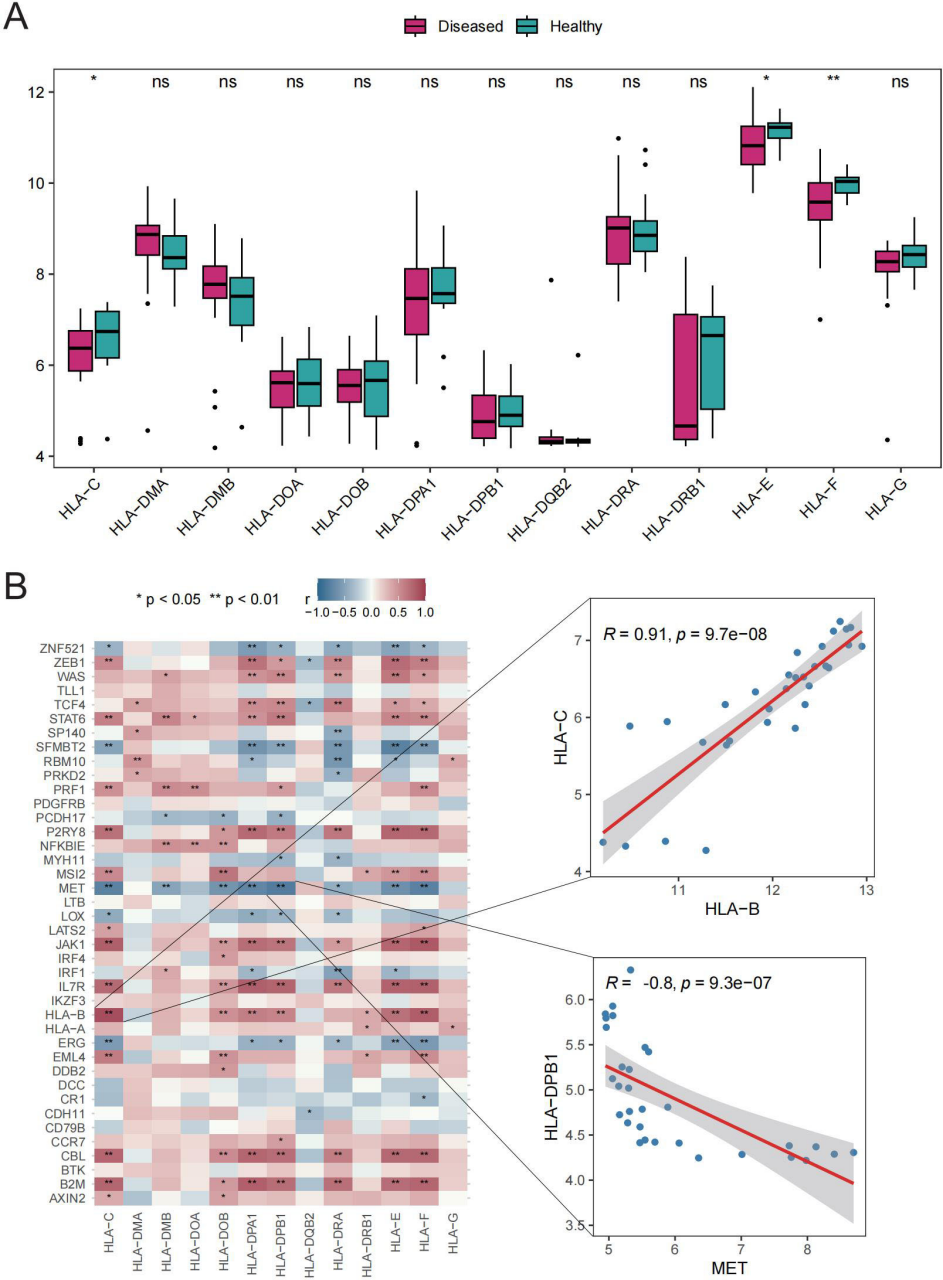
HLA gene analysis. A: Box plot showing gene expression differences between the HC and GD groups. B: Spearman correlation of Tex-related gene expression in GD samples. ns (*p*>0.05), *P<0.05, **P<0.01.

**Figure 3:**
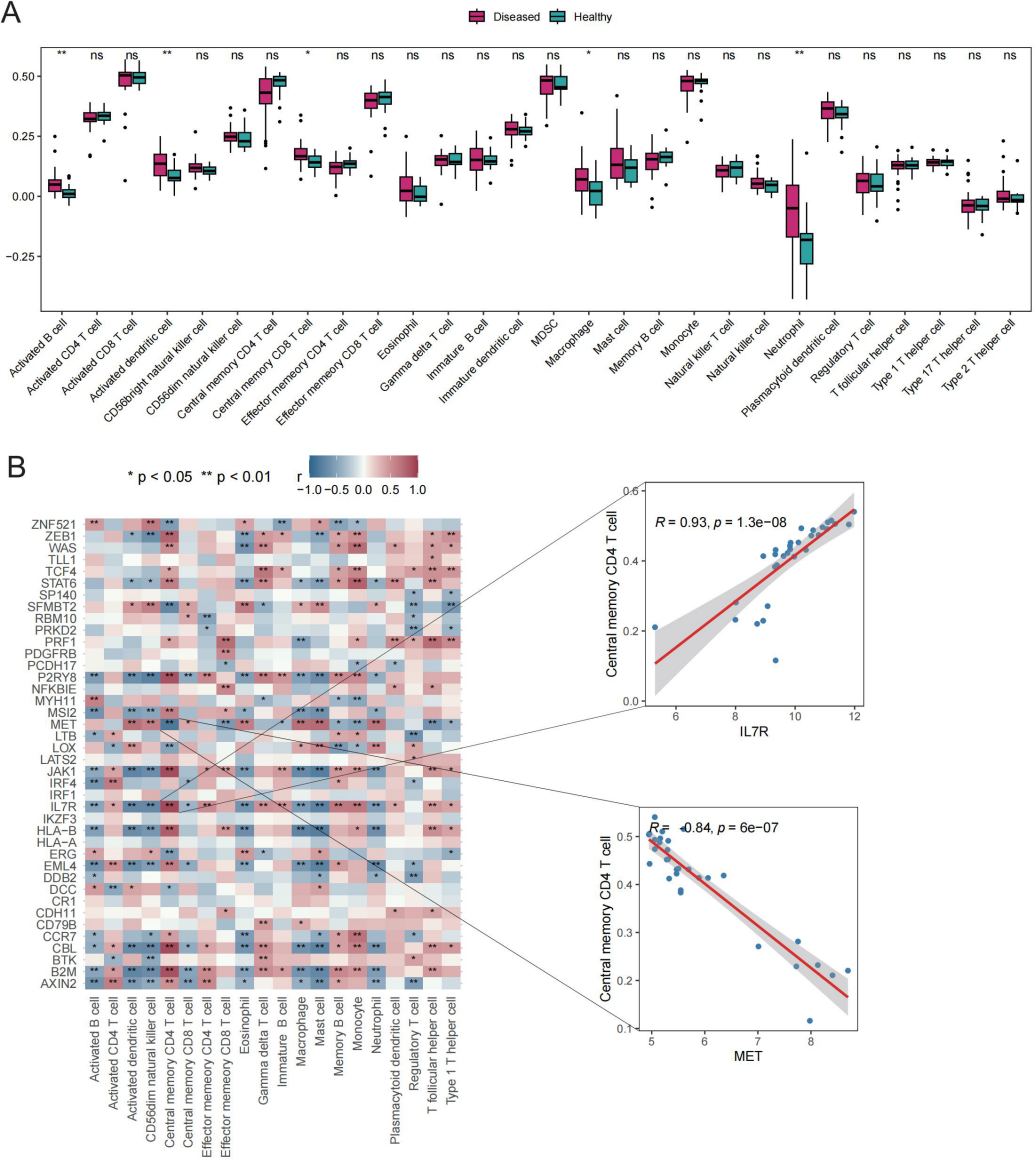
Immune cell infiltration scores. A: Box plot depicting expression of top 20 genes in the HC vs. GD groups. B: Spearman correlation of gene expression related to Tex in GD samples. ns (*p*>0.05), *P<0.05, **P<0.01.

The potential roles of Tex-related genes in the pathogenesis of GD were investigated through a systematic analysis. Initial comparisons focused on HLA gene expression, immune cell infiltration scores, and immune response scores in different normal and GD patient samples. Spearman correlation analyses were performed to assess the relationships of HLA gene expression, immune cell infiltration scores, and immune response scores with Tex-related gene expression in GD patient samples. The results revealed down-regulation of three HLA genes (*HLA-C*, *HLA-E*, and *HLA-F*) in GD patient samples (**Figure 2A**). Further analyses demonstrated significant correlations between the expression of most aberrantly expressed HLA genes in GD and Tex-related genes (**Figure 2B**). Compared with HC samples, GD patient samples exhibited significantly higher infiltration scores for five immune cell types, specifically activated B cells, activated dendritic cells, central memory CD8 T cells, macrophages, and neutrophils (**Figure 3A**). Substantial correlations were observed between the immune cell infiltration scores in GD patient samples and the expression levels of Tex-associated genes. Notably, *CBL*, a Tex-related gene found to be differentially expressed in GD, showed a significant negative correlation with immune cell enrichment in GD (**Figure 3B**).

**Figure 4:**
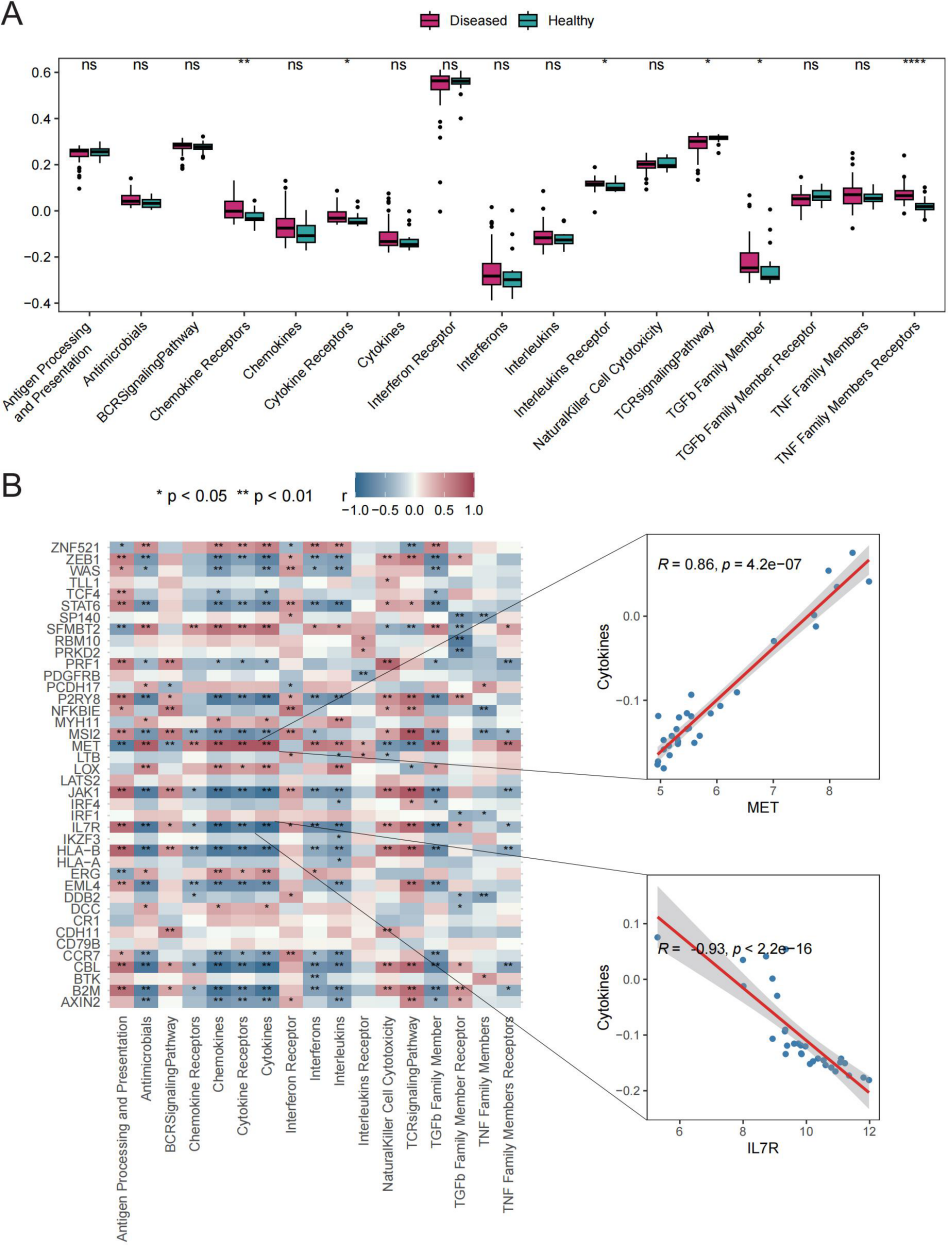
Immune response scores. A: Box plot showing differences in immune response scores between the HC and GD groups. B: Spearman correlation analysis for Tex-related gene expression in GD samples. ns (*p*>0.05), *P<0.05, **P<0.01, ****P<0.0001.

Six immune response scores, including those for Chemokine Receptors, Cytokine Receptors, Interleukin Receptors, TGFβ Family Members, and TNF Family Members Receptors, were significantly different between GD patient samples and HC samples (**Figure 4A**). Significant correlations were found between immune response scores and the expression of Tex-related genes. Of particular interest was a close correlation between *CBL* expression and the significantly enriched immune responses in samples from GD patients (**Figure 4B**). These results suggest that the expression of Tex-related genes may impact GD progression through immunomodulatory effects.

### Classification of GD subtypes based on Tex-related gene expression

**Figure 5:**
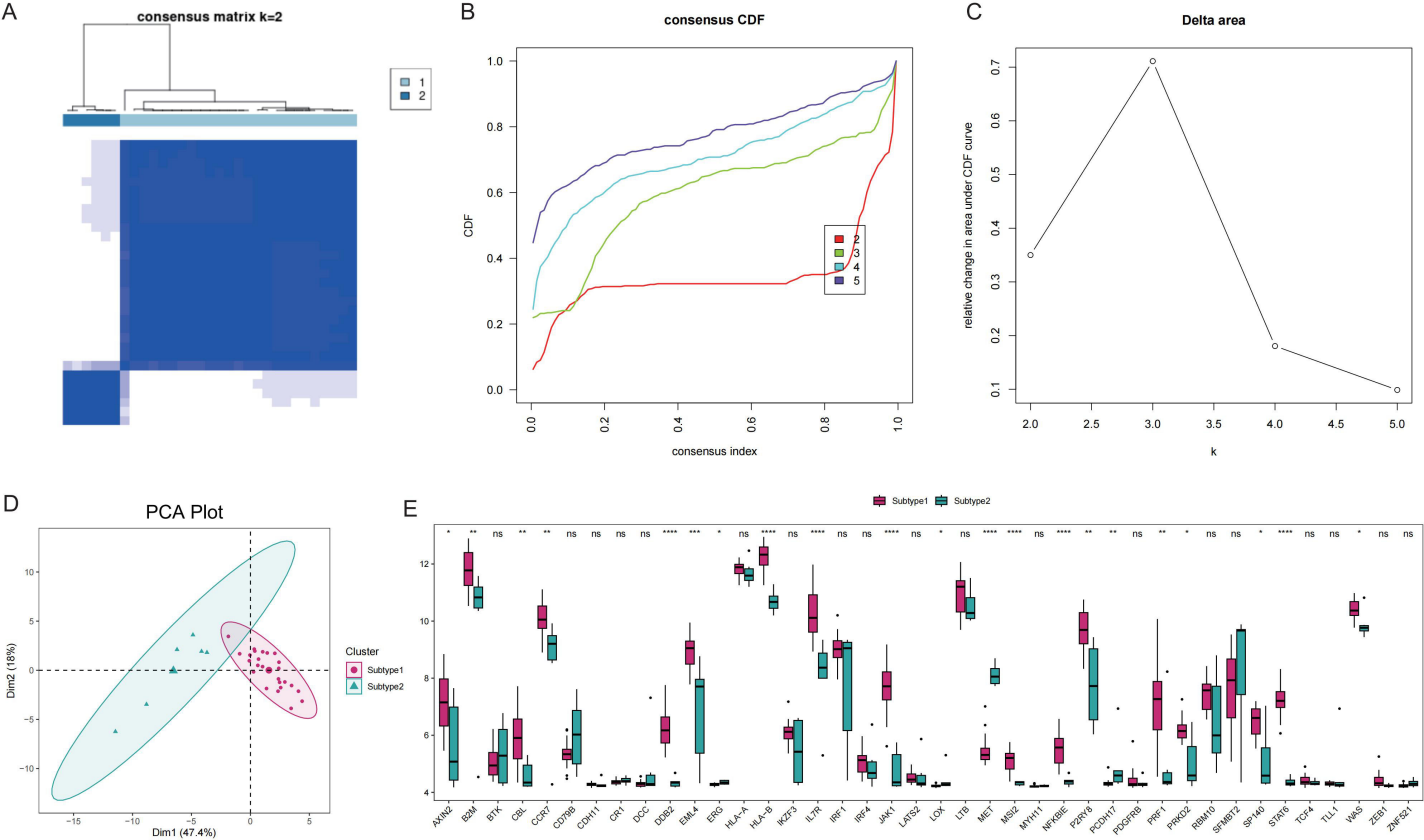
Disease subtype analysis. A: Consistent clustering of Tex-related genes; Subtype1 and Subtype2 represent two subgroups. B: Cumulative distribution function (CDF) plot; C: Delta Area Plot; D: Principal component analysis of T cell exhaustion-related genes; E: Box plots of Tex-related gene expression in the two subtypes. ns (*p*>0.05), *P<0.05, **P<0.01, ***P<0.001, and ****P<0.0001.

To elucidate the expression patterns of Tex-related genes associated with GD, we performed unsupervised cluster analysis, which classified 31 GD patient samples into two subtypes: Subtype1 (n=25) and Subtype2 (n=6), based on the expression profiles of 40 Tex-related genes (**Figures 5A-C**). The two disease subtypes were effectively determined by expression of T cell depletion-related genes (**Figure 5D**), with 21 Tex-related genes showing significantly differential expression, including notably *CBL*, which exhibited abnormally high expression in Subtype1 (**Figure 5E**).

**Figure 6:**
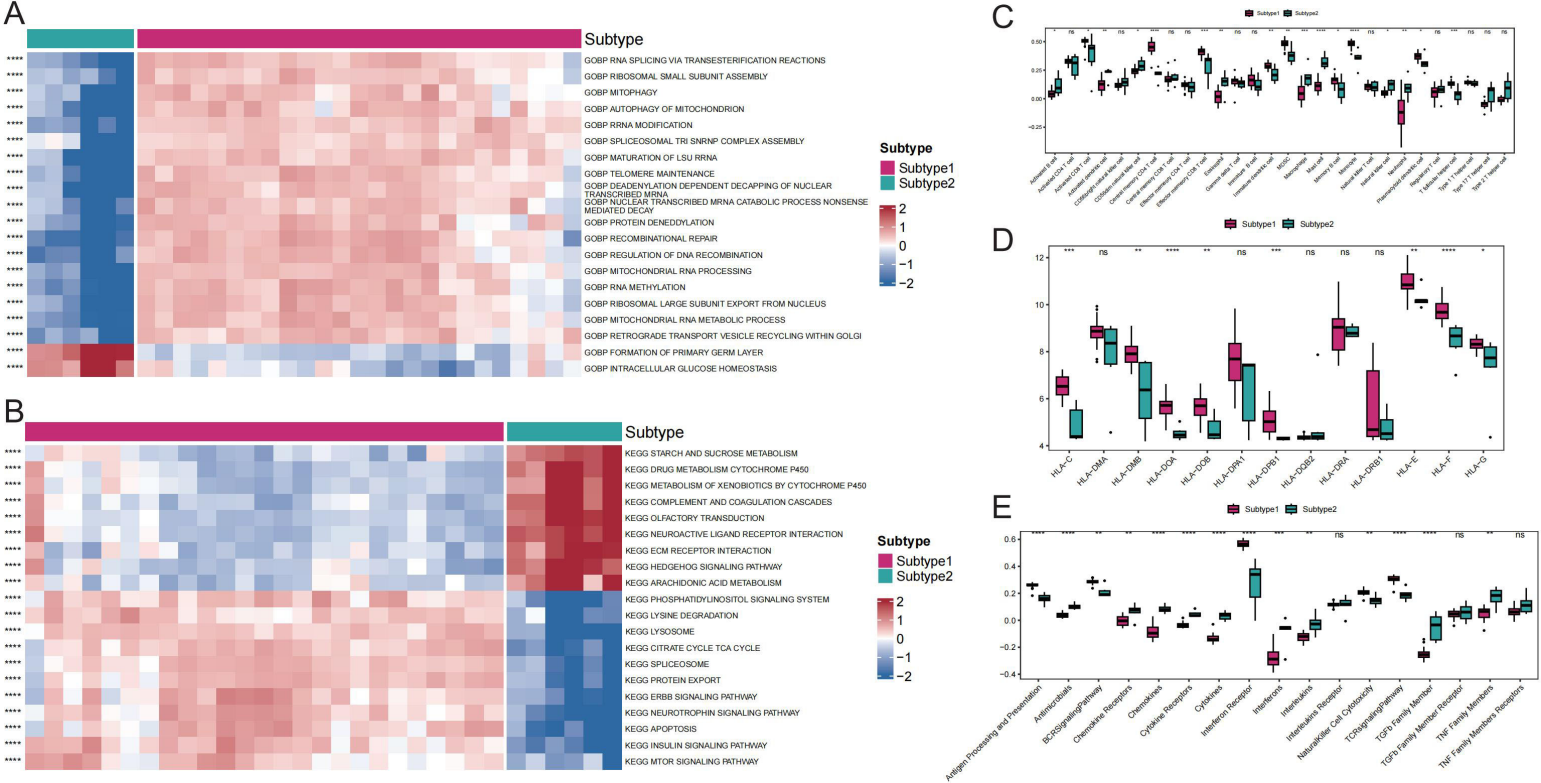
Comparison of immune responses between subtypes of GD. A: Heat map of differential gene ontology biological process (GOBP) pathway activity between GD subtypes. B: Heat map of differential Kyoto Encyclopedia of Genes and Genomes (KEGG) pathway activity between GD subtypes. C: Differential immune cell infiltration scores between GD subtypes. D: Comparison of HLA gene expression between GD subtypes. E: Differential immunoreactivity scores between GD subtypes. ns (*p*>0.05), *P<0.05, **P<0.01, ***P<0.001, and ****P<0.0001.

Comparisons between subtypes revealed differences in pathway activity, HLA gene expression, immune cell infiltration score, and immune response score. Specifically, the results revealed significant distinctions in the pathway activities of the ECM RECEPTOR INTERACTION, ERBB SIGNALING PATHWAY, and MTOR SIGNALING PATHWAY between the subtypes (**Figures 6A, B**). Additionally, 17 immune cell infiltration scores, including those for activated B cells, activated dendritic cells, and activated CD8+ T cells, differed significantly between the subtypes (**Figure 6C**). The expression of 8 HLA genes and immune response scores related to cytokine receptors, natural killer cells, cytotoxicity, and the T-cell receptor (TCR) signaling pathway also showed significant differences between the two subtypes (**Figures 6D, E**). These findings suggest the presence of two distinct disease subtypes within GD that have divergent immunological characteristics based on the distinct expression patterns of Tex-related genes.

### Identification of potential therapeutic drugs through WGCNA and subtype hub DEG analysis

**Figure 7:**
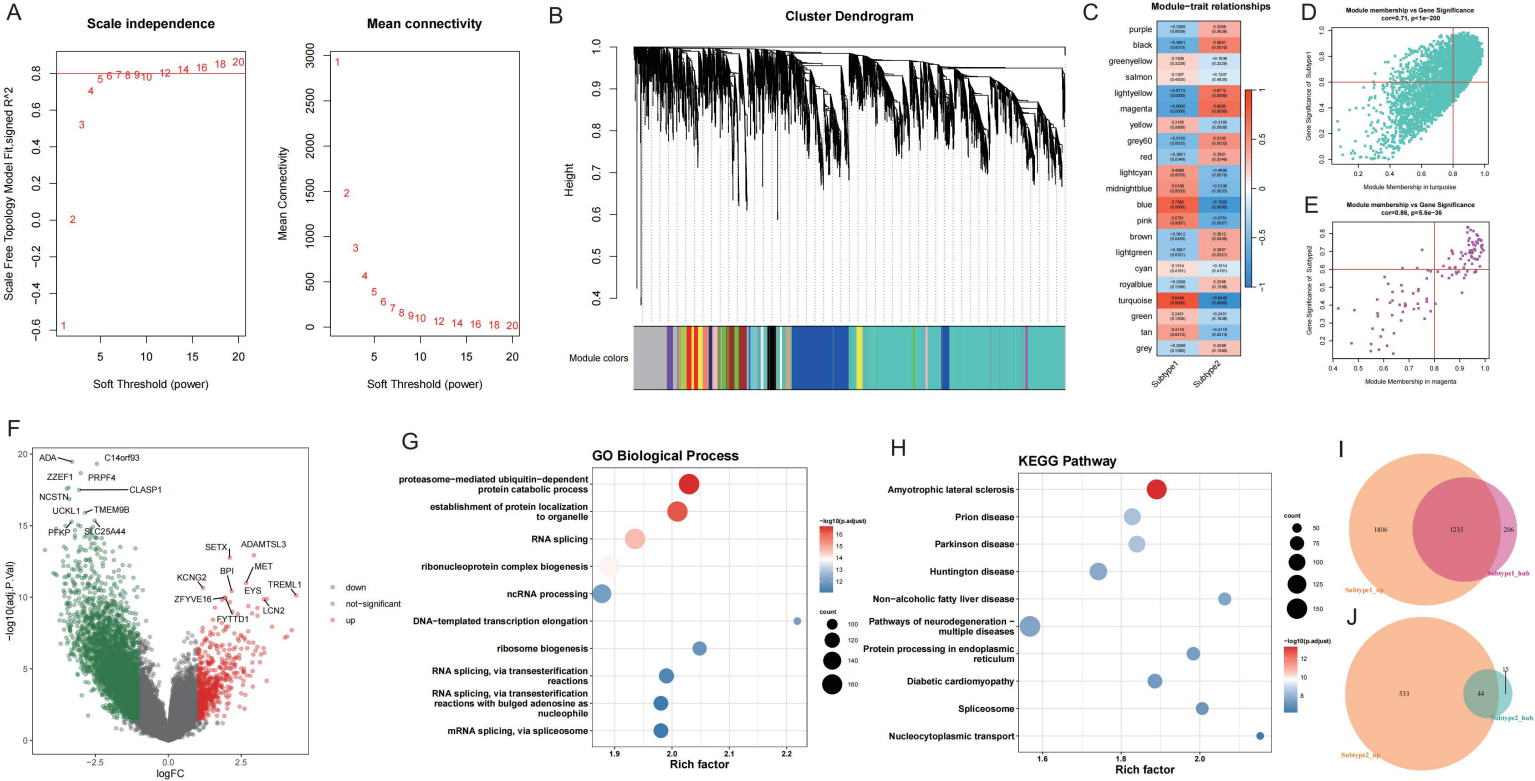
WGCNA screening of core genes for key modules of each GD subtype. A: Analysis of network topology for various soft-thresholding powers. B: Gene dendrogram and module colors. C: Module-feature correlation. D-E: Turquoise and magenta module screening for core genes. F: Volcano map of differentially expressed genes between GD subtypes; Subtype1 is control. G-H: Enrichment analysis of differentially expressed genes between GD subtypes. I-J: Wayne's diagram of core genes and upregulated genes in each subtype.

To explore key molecules associated with the GD subtypes, WGCNA was performed on the top 50% of genes and identified 21 modules (**Figures 7A, B**). Based on the calculated correlation coefficient, the turquoise module was most positively correlated with Subtype1, and the magenta module was most positively correlated with Subtype2 (**Figure 7C**). These modules were considered key modules for Subtype1 and Subtype2, respectively, and 1441 and 59 module core genes were screened based on GS and MM, respectively (**Figures 7D, E**).

Upon application of the limma package, 3041 DEGs with upregulated expression in Subtype1 and 577 DEGs with upregulated expression in Subtype2 were identified (**Figure 7F**). Enrichment analysis was conducted on these DEGs (**Figures 7G, H**), and the intersection of core genes with upregulated genes in each subtype yielded 1235 core DEGs in Subtype1 (**Figure 7I**) and 44 core DEGs in Subtype2 (**Figure 7J**).

**Figure 8:**
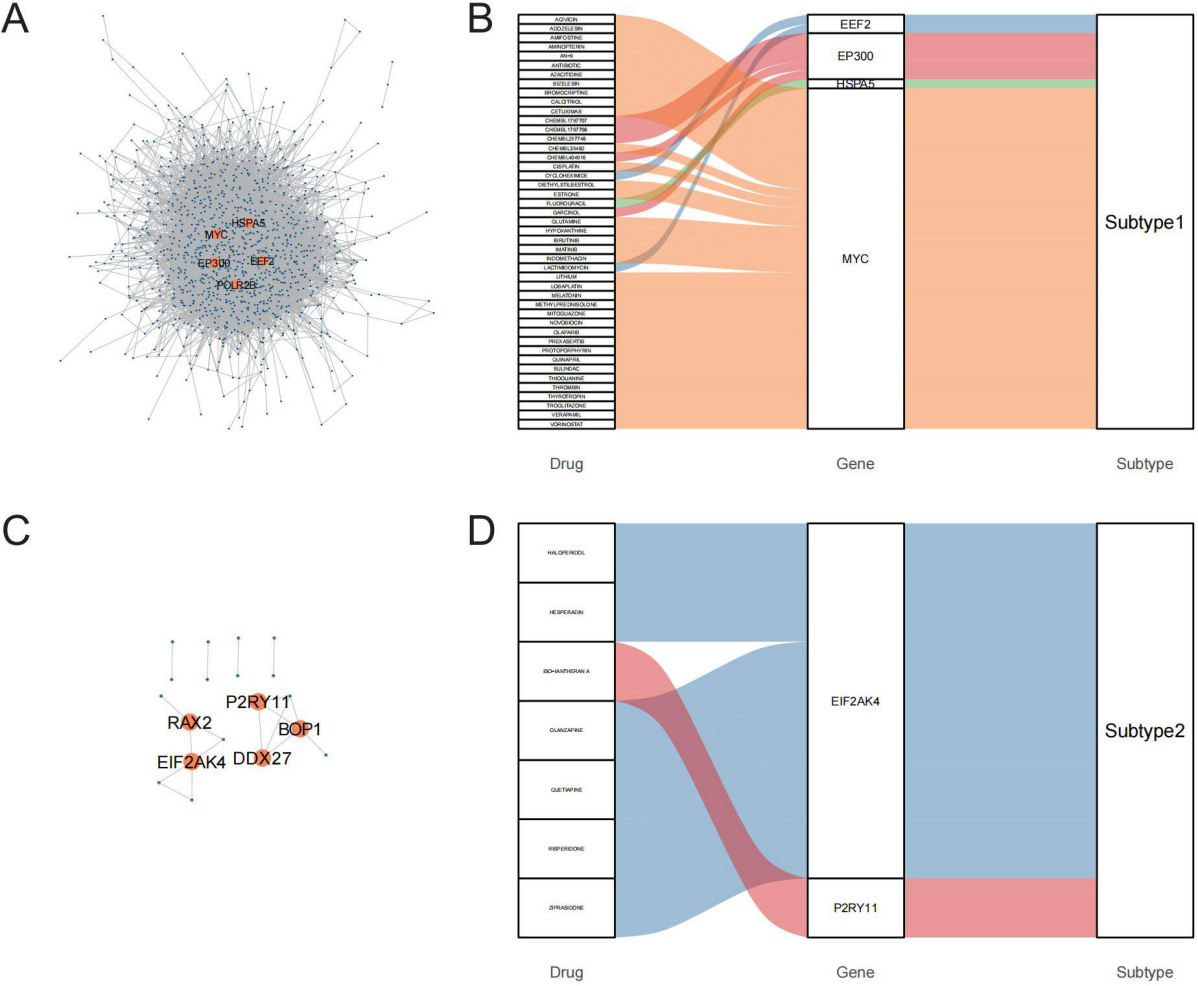
PPI networks identify hub genes for mining of potential drugs. A-C: PPI networks of core differentially expressed genes (DEGs) between the GD subtypes; A is Subtype1, C is Subtype2. B-D: Interactions of hub genes of GD subtypes with drugs.

To identify potential therapeutic targets and drugs, subtype hub genes and drug molecules were screened using the PPI network and DGIdb database. From the constructed PPI networks for Subtype1 and Subtype2, the top 5 hub genes were selected based on node connectivity (**Figures 8A, C**). The hub genes for Subtype1 were *HSPA5*, *MYC*, *EEF2*, *EP300*, and *POLR2B*, while those for Subtype2 were *RAX2*, *P2R2B*, and *P2R2B*. Queries of interactions between hub genes and drugs in the DGIdb database revealed interactions of *MYC* and *EIF2AK4* with various drugs (**Figures 8B, D**). Examples inclu*de novo*biocin, glutamine, and thioguanine for *MYC* and haloperidol, Hesperadin, and quetiapine for *EIF2AK4*.

### CBL mRNA expression in PBMCs of GD patients

**Figure 9:**
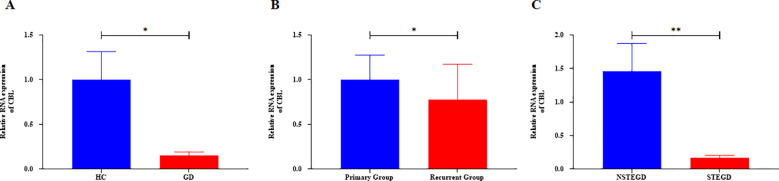
Expression of Tex-related gene *CBL* in PBMCs of GD patients. A: Abnormal downregulation of *CBL* mRNA expression in GD patients. B: Lower *CBL* mRNA expression in the recurrent group compared with the primary group. C: Lower *CBL* mRNA expression in GD patients with moderate-to-severe thyroid enlargement compared to those without such enlargement. The error bars presented on multiple panels represent the Standard Error of the Mean (SEM), *P<0.05, **P<0.01. GD: Graves' disease patient group, HC: healthy control group, NSTEGD: those without thyroid enlargement or with Grade I enlargement GD, STEGD: those with Grade II-III enlargement GD.

Based on the general association of Tex with a favorable prognosis in autoimmune diseases (7), we examined whether Tex-related driver genes are abnormally downregulated in GD patient samples. Considering the results of a literature review, the *CBL* gene was chosen for validation of its expression in GD patient-derived PBMCs. *CBL* mRNA demonstrated abnormally downregulated expression in GD patients (**Figure 9A**, *P*=0.043). Furthermore, compared with that in samples from the primary group, *CBL* mRNA expression was lower in samples from the recurrent group (**Figure 9B**, *P*=0.020). *CBL* mRNA expression was significantly lower in GD patients with moderate-to-severe thyroid enlargement than in those without such enlargement (**Figure 9C**, *P*=0.002). Correlation analysis revealed a negative correlation between *CBL* mRNA expression and TRAb levels in GD patients. However, no significant associations were observed between *CBL* expression and the levels of FT3, FT4, and TSH (**Table 1**). These findings suggest that the Tex-related gene *CBL* may play a crucial immunoregulatory role in the pathogenesis of GD.

**Table 1:** Correlation analysis between CBL expression and laboratory indicators.

Variable	r	*P*
FT3 (pmol/L)	-0.05	0.563
FT4 (pmol/L)	0.01	0.924
TSH (mIU/L)	0.02	0.833
TRAb (IU/ml)	-0.29	0.002**

**P <0.01.

## Discussion

GD is an autoimmune thyroid disorder characterized by elevated TRAb levels, lymphocytic infiltration, and tissue hyperactivation, with effects on multiple systems (2). T-cell dysfunction is pivotal in GD pathogenesis and progression, and Tex, a distinct T-cell functional state, has been implicated in various autoimmune disorders (15). This study explored alterations of Tex-related gene expression in GD, and the results provide insight on the Tex phenotype and potential regulatory mechanisms. By identifying hub genes associated with different subtypes of GD, potential therapeutic avenues are revealed, offering theoretical support and guiding future investigations into the roles of Tex-related genes in GD pathogenesis.

In autoimmune diseases, a complex interplay of factors, including prolonged antigenic stimulation, restricted CD4+ T-cell responses, limited co-stimulation during initial T-cell activation, diminished gamma-chain cytokine signaling, and characteristics of the tissue microenvironment (e.g., hypoxia, nutrient deficiencies, pH abnormalities), contributes to T-cell differentiation along exhaustion pathways (16). Tex results in substantial alterations in effector function, cytokine responses, and metabolic profiles, accompanied by specific transcriptional changes (17). Recent investigations have identified Tex in several autoimmune diseases, which exhibit abnormal expression of inhibitory receptors and altered gene profiles. For instance, Tex phenotype manifestation has been observed in pancreatic islet-specific CD8 T cells in type 1 diabetes (18). Frenz et al. demonstrated increased expression of inhibitory receptors programmed cell death protein 1 (PD-1) and cytotoxic T-lymphocyte associated protein 4 (CTLA-4) in CD4+ T cells from RA patients (19), while another study in ANCA-associated vasculitis patients revealed dysregulated expression of Tex-related genes in CD8+ T cells (20). Additionally, A et al. reported a depleted T regulatory cell 2 (Treg2) cell phenotype and elevated expression of Tex-related genes in SLE patients (21). The present investigation aimed to elucidate the intricate interactions of Tex-related genes in GD, and the results reveal aberrant expression of Tex-related genes in PBMCs of GD patients, suggesting a potential role for Tex in GD pathogenesis.

Marked by the expression of inhibitory receptors like programmed death ligand 1 (PD-L1), lymphocyte activation gene 3 (LAG-3), T-cell immunoglobulin and mucin domain 3 (TIM-3), cluster of differentiation 244 (CD244), CD160, and T cell immunoglobulin and ITIM domain (TIGIT), crucially modulate autoimmune responses, negatively regulating immunopathological pathways through adjustments in their transcriptional expression profiles (8). Tex is integral to maintaining immune homeostasis, although it does not alter the initial disease progression direction. For example, the elevated PD-L1 expression in synovial tissue and fluid of RA patients suggests the key role of the PD-1/PD-L1 co-inhibitory pathway in immune homeostasis regulation in RA (22). In Crohn’s disease, functional enrichment of Tex-related genes indicates their significance in various lymphocyte and immune activation pathways. Conversely, in SLE patients, aberrant expression of Tex-related genes in Tregs is associated with the nuclear factor kappa B (NF-κB) signaling pathway (21). These insights underscore the pivotal regulatory role of Tex in diverse autoimmune diseases. Previous studies implicated activated dendritic cells, macrophages, and neutrophils in the autoimmune and inflammatory responses of GD. These cells contribute through antigen presentation, cytokine secretion, and inflammatory mediator release. Furthermore, B cells activated by helper CD4+ T cells produce autoantibodies, intensifying the autoimmune response (23). Our study reveals that Tex-related gene expression in GD patients may regulate the recruitment of immune cells, such as activated dendritic cells, macrophages, neutrophils, and activated B cells, thereby participating in chemokine and cytokine receptor-mediated immune responses. Accordingly, we hypothesize that aberrantly expressed Tex-related genes in GD may influence disease development and progression by modulating the GD immune response. However, further functional experimental studies are required to confirm this hypothesis.

In our comprehensive exploration of the relationship between clinical phenotypes and Tex-related genes in GD patients, unsupervised cluster analysis revealed 40 Tex-related genes capable of classifying sequenced GD cases into two subtypes. These subtypes exhibited significant disparities in gene expression, immune cell infiltration, and immune response enrichment, consistent with previous findings (21). These findings suggest the involvement of Tex-related genes in the phenotypic variation of GD patients through immunomodulatory functions. Furthermore, through the identification of core genes expressed in different GD subtypes, we pinpointed potential therapeutic agents such as novobiocin, glutamine, thioguanine, haloperidol, Hesperadin, and quetiapine. Specifically, glutamine, which is known for its immune-modulating properties, has demonstrated modulatory effects in RA (24). Thioguanin, a nucleoside analog widely used in immunomodulatory therapies, has applications in treating Crohn’s disease, autoimmune hepatitis, lupus nephritis, and transplant rejection prevention (25). Haloperidol, an antipsychotic, exhibits a potential modulating effect on pro-inflammatory T-cell function and has shown therapeutic efficacy in a rat model of RA, despite being primarily indicated for psychiatric disorders (26). Hesperadin, an antioxidant, has demonstrated efficacy in treating RA and experimental allergic encephalomyelitis (27). Quetiapine has been reported to reduce autoimmune-mediated demyelination by inhibiting T-cell responses (28). While the use of these drugs in GD remains unreported, drawing from previous studies, we posit that these drugs targeting Tex-associated genes may present novel avenues for GD treatment.

For further analysis in GD patients, *CBL* was selected as a candidate gene due to its abnormally low expression in GD samples and its role in negatively regulating the immune infiltration of various cell types and immune responses. RT-qPCR analysis confirmed the abnormally low expression of *CBL* in PBMCs of GD patients, consistent with microarray sequencing results, and revealed a negative correlation with the level of TRAb—a crucial diagnostic indicator for GD involving the aberrant activation of pro-inflammatory lymphocytes, including T cells. These findings support the results of our comprehensive bioinformatics analysis, suggesting a potential role for the Tex-related gene *CBL* in the abnormal immune regulation of GD. When GD cases were categorized into primary and relapse groups, significantly lower *CBL* mRNA expression was observed in the relapse group. In patients with newly diagnosed GD, clinical phenotypes can be separated according to the presence of moderate-to-severe thyroid enlargement and the absence of such enlargement. We found that *CBL* mRNA expression was significantly downregulated in the former group. *CBL* encodes an E3 ubiquitin-protein ligase, acting as a negative regulator in various signaling pathways (29). Previous studies have reported a pro-Tex role for *CBL* in diseases such as esophageal cancer, in which *CBL* interacts with SPRY1 to promote Tex, contributing a pro-cancer effect. Additionally, studies on *CBLB*, a closely related homolog of *CBL*, have demonstrated its promotion of Tex (30). Therefore, we hypothesize that the low expression of *CBL* in GD patients may promote GD relapse and thyroid enlargement by inhibiting Tex. However, mechanistic studies are required to further validate this hypothesis.

Our study has some limitations. Our findings were primarily derived from comprehensive bioinformatics analyses, and initial validation through RT-qPCR was conducted for only one Tex-related gene. Additionally, the small sample size necessitates additional functional experimental validation and expansion of the clinical sample size. These steps are crucial for a comprehensive assessment of the exact role of Tex-related genes in the pathogenesis and prognosis of GD. Recent advancements in technologies such as single-cell sequencing, multiparameter flow cytometry, and high-dimensional mass cytometry offer opportunities to explore T-cell heterogeneity, various phenotypes, functions, and transcriptional programs. Our group plans to conduct in-depth studies in these areas for a more nuanced understanding of the role of Tex in GD.

## Conclusions

In summary, Tex may be integral to the pathogenesis and prognostic mechanisms of GD. Our study provides initial insights, indicating that the Tex-related gene *CBL* is associated with immunomodulatory functions and holds promise as a prognostic biomarker for disease relapse. However, this finding requires further validation in more comprehensive studies.

## Data Availability

The original contributions presented in the study are included in the article/**Supplementary Materials**. Further inquiries can be directed to the corresponding author.

## References

[1] Smith TJ Hegedüs L. Graves' Disease. N Engl J Med. (2016) 375:1552–65. doi: 10.1056/NEJMra1510030 27797318

[2] Antonelli A Fallahi P Elia G Ragusa F Paparo SR Ruffilli I. Graves' disease: Clinical manifestations, immune pathogenesis (cytokines and chemokines) and therapy. Best Pract Res Clin Endocrinol Metab. (2020) 34:101388. doi: 10.1016/j.beem.2020.101388 32059832

[3] Hansen M Cheever A Weber KS O'Neill KL. Characterizing the interplay of lymphocytes in graves' Disease. Int J Mol Sci. (2023) 24. doi: 10.3390/ijms24076835 PMC10094834 37047805

[4] Chopp L Redmond C O'Shea JJ Schwartz DM. From thymus to tissues and tumors: A review of T-cell biology. J Allergy Clin Immunol. (2023) 151:81–97. doi: 10.1016/j.jaci.2022.10.011 36272581 PMC9825672

[5] Álvarez-Sierra D Marín-Sánchez A Gómez-Brey A Bello I Caubet E Moreno-Llorente P. Lymphocytic thyroiditis transcriptomic profiles support the role of checkpoint pathways and B cells in pathogenesis. Thyroid. (2022) 32:682–93. doi: 10.1089/thy.2021.0694 PMC9360182 35403441

[6] Wherry EJ Kurachi M. Molecular and cellular insights into T cell exhaustion. Nat Rev Immunol. (2015) 15:486–99. doi: 10.1038/nri3862 PMC4889009 26205583

[7] Osum KC Burrack AL Martinov T Sahli NL Mitchell JS Tucker CG. Interferon-gamma drives programmed death-ligand 1 expression on islet β cells to limit T cell function during autoimmune diabetes. Sci Rep. (2018) 8:8295. doi: 10.1038/s41598-018-26471-9 29844327 PMC5974126

[8] Collier JL Weiss SA Pauken KE Sen DR Sharpe AH. Not-so-opposite ends of the spectrum: CD8(+) T cell dysfunction across chronic infection, cancer and autoimmunity. Nat Immunol. (2021) 22:809–19. doi: 10.1038/s41590-021-00949-7 PMC9197228 34140679

[9] Limbach M Saare M Tserel L Kisand K Eglit T Sauer S. Epigenetic profiling in CD4+ and CD8+ T cells from Graves' disease patients reveals changes in genes associated with T cell receptor signaling. J Autoimmun. (2016) 67:46–56. doi: 10.1016/j.jaut.2015.09.006 26459776

[10] Zhang Y Wei J Zhou H Li B Chen Y Qian F. Identification of two potential immune-related biomarkers of Graves' disease based on integrated bioinformatics analyses. Endocrine. (2022) 78:306–14. doi: 10.1007/s12020-022-03156-y 35962894

[11] Zhang Z Chen L Chen H Zhao J Li K Sun J. Pan-cancer landscape of T-cell exhaustion heterogeneity within the tumor microenvironment revealed a progressive roadmap of hierarchical dysfunction associated with prognosis and therapeutic efficacy. EBioMedicine. (2022) 83:104207. doi: 10.1016/j.ebiom.2022.104207 35961204 PMC9382263

[12] Zhang X Zhang S Yan X Shan Y Liu L Zhou J. m6A regulator-mediated RNA methylation modification patterns are involved in immune microenvironment regulation of periodontitis. J Cell Mol Med. (2021) 25:3634–45. doi: 10.1111/jcmm.16469 PMC8034465 33724691

[13] Ross DS Burch HB Cooper DS Greenlee MC Laurberg P Maia AL. 2016 american thyroid association guidelines for diagnosis and management of hyperthyroidism and other causes of thyrotoxicosis. Thyroid. (2016) 26:1343–421. doi: 10.1089/thy.2016.0229 27521067

[14] Cho YY Chung YJ. Vitamin D supplementation does not prevent the recurrence of Graves' disease. Sci Rep. (2020) 10:16. doi: 10.1038/s41598-019-55107-9 31913301 PMC6949266

[15] Belk JA Daniel B Satpathy AT. Epigenetic regulation of T cell exhaustion. Nat Immunol. (2022) 23:848–60. doi: 10.1038/s41590-022-01224-z PMC10439681 35624210

[16] Ogura H Preston-Hurlburt P Perdigoto AL Amodio M Krishnaswamy S Clark P. Identification and analysis of islet antigen-specific CD8(+) T cells with T cell libraries. J Immunol. (2018) 201:1662–70. doi: 10.4049/jimmunol.1800267 PMC6449153 30082321

[17] Laban S Suwandi JS Unen van V Pool J Wesselius J Höllt T. Heterogeneity of circulating CD8 T-cells specific to islet, neo-antigen and virus in patients with type 1 diabetes mellitus. PloS One. (2018) 13:e0200818. doi: 10.1371/journal.pone.0200818 30089176 PMC6082515

[18] Linsley PS Long SA. Enforcing the checkpoints: harnessing T-cell exhaustion for therapy of T1D. Curr Opin Endocrinol Diabetes Obes. (2019) 26:213–8. doi: 10.1097/MED.0000000000000488 PMC6635062 31157632

[19] Frenz T Grabski E Buschjäger D Vaas LA Burgdorf N Schmidt RE. CD4(+) T cells in patients with chronic inflammatory rheumatic disorders show distinct levels of exhaustion. J Allergy Clin Immunol. (2016) 138:586–589.e10. doi: 10.1016/j.jaci.2016.04.013 27264455

[20] McKinney EF Lee JC Jayne DR Lyons PA Smith KG. T-cell exhaustion, co-stimulation and clinical outcome in autoimmunity and infection. Nature. (2015) 523:612–6. doi: 10.1038/nature14468 PMC4623162 26123020

[21] Guo C Liu Q Zong D Zhang W Zuo Z Yu Q. Single-cell transcriptome profiling and chromatin accessibility reveal an exhausted regulatory CD4+ T cell subset in systemic lupus erythematosus. Cell Rep. (2022) 41:111606. doi: 10.1016/j.celrep.2022.111606 36351407

[22] Globig AM Mayer LS Heeg M Andrieux G Ku M Otto-Mora P. Exhaustion of CD39-expressing CD8(+) T cells in crohn's disease is linked to clinical outcome. Gastroenterology. (2022) 163:965–981.e31. doi: 10.1053/j.gastro.2022.06.045 35738329

[23] Purnamasari D Soewondo P Djauzi S. Dendritic cells in Graves' disease. Acta Med Indones. (2015) 47:61–9. 25948770

[24] Nakaya M Xiao Y Zhou X Chang JH Chang M Cheng X. Inflammatory T cell responses rely on amino acid transporter ASCT2 facilitation of glutamine uptake and mTORC1 kinase activation. Immunity. (2014) 40:692–705. doi: 10.1016/j.immuni.2014.04.007 24792914 PMC4074507

[25] Legué C Legros L Kammerer-Jacquet S Jézequel C Houssel-Debry P Uguen T. Safety and efficacy of 6-thioguanine as a second-line treatment for autoimmune hepatitis. Clin Gastroenterol Hepatol. (2018) 16:290–1. doi: 10.1016/j.cgh.2017.07.032 28782666

[26] Fahmy Wahba MG Shehata Messiha BA Abo-Saif AA. Ramipril and haloperidol as promising approaches in managing rheumatoid arthritis in rats. Eur J Pharmacol. (2015) 765:307–15. doi: 10.1016/j.ejphar.2015.08.026 26302059

[27] Qi W Lin C Fan K Chen Z Liu L Feng X. Hesperidin inhibits synovial cell inflammation and macrophage polarization through suppression of the PI3K/AKT pathway in complete Freund's adjuvant-induced arthritis in mice. Chem Biol Interact. (2019) 306:19–28. doi: 10.1016/j.cbi.2019.04.002 30954464

[28] Mei F Guo S He Y Wang L Wang H Niu J. Quetiapine, an atypical antipsychotic, is protective against autoimmune-mediated demyelination by inhibiting effector T cell proliferation. PloS One. (2012) 7:e42746. doi: 10.1371/journal.pone.0042746 22912731 PMC3418290

[29] Lock P S.T. I Straffon AF Schieb H Hovens CM Stylli SS. Spred-2 steady-state levels are regulated by phosphorylation and Cbl-mediated ubiquitination. Biochem Biophys Res Commun. (2006) 351:1018–23. doi: 10.1016/j.bbrc.2006.10.150 17094949

[30] Kumar J Kumar R Singh Kumar A Tsakem EL Kathania M Riese MJ. Deletion of Cbl-b inhibits CD8(+) T-cell exhaustion and promotes CAR T-cell function. J Immunother Cancer. (2021) 9. doi: 10.1136/jitc-2020-001688 PMC7813298 33462140

