# Supplementary material for: T-cell exhaustion-related genes in Graves’ disease: a comprehensive genome mapping analysis
Source: Front Endocrinol (Lausanne). 2024 Aug 22;15:1364782. doi: 10.3389/fendo.2024.1364782 (PMC11374593; doi:10.3389/fendo.2024.1364782)
Supplement: Supplementary file 1 [file Table1.docx]

**Table S1 Basic information of the study -** **RT-qPCR**

|  | HC | GD | | *P* |
| --- | --- | --- | --- | --- |
| Number | 47 | | 112 | - |
| Male | 10 | | 33 | - |
| Female | 37 | | 79 | - |
| year | 38.40±14.44 | | 38.09±11.37 | 0.94 |
| FT3 (pmol/L) | 4.94(4.48-5.30) | | 13.82 (8.82-23.43) | <0.0001^****^ |
| FT4 (pmol/L) | 16.00(14.37-17.40) | | 38.79(23.40-61.16) | <0.0001^****^ |
| TSH (mIU/L) | 1.80(1.27-2.44) | | 0.005 (0.005, 0.005) | <0.0001^****^ |
| TRAb (IU/L) | 0.80 (0.80-1.11) | | 15.66(7.89-32.62) | <0.0001^****^ |
| FT3: Free T3, FT4: Free T4, TRAb: Thyroid Stimulating Hormone Receptor Antibody, ^****^*P* <0.0001: Indicates a highly significant statistical result. | | | | |
