# Supplementary material for: T-cell exhaustion-related genes in Graves’ disease: a comprehensive genome mapping analysis
Source: Front Endocrinol (Lausanne). 2024 Aug 22;15:1364782. doi: 10.3389/fendo.2024.1364782 (PMC11374593; doi:10.3389/fendo.2024.1364782)
Supplement: Supplementary file 2 [file Table2.docx]

| **Table S2 Baseline characteristics of patients with GD** | | | | | | | |
| --- | --- | --- | --- | --- | --- | --- | --- |
| Group | Phenotype | Number | Female (%) | FT3 (pmol/L) | FT4 (pmol/L) | TSH (mIU/L) | TRAb (IU/L) |
| Primary |  | 70 | 75.71 | 13.78(8.91,23.54) | 39.55(23.59,55.15) | 0.005 (0.005,0.005) | 13.86 (7.88, 30.76) |
|  | NSTEGD | 45 | 77.78 | 14.04(9.24,30.80) | 43.60(30.42,69.24) | 0.005 (0.005,0.005) | 11.57(7.865,27.41) |
|  | STEGD | 25 | 72 | 11.52(7.05,19.27) | 35.58(14.92,47.88) | 0.005 (0.005,0.005) | 25.47(8.800,33.44) |
| Recurrent |  | 42 | 61.90 | 14.89(7.98,23.46) | 36.41(19.91,66.72) | 0.005 (0.005,0.006) | 17.66 (7.58, 36.84) |
| FT3: Free T3, FT4: Free T4, TRAb: Thyroid Stimulating Hormone Receptor Antibody, NSTEGD (Non-Severe Thyroid Enlargement Graves' Disease): Patients without thyroid enlargement or with Grade I thyroid enlargement. STEGD (Severe Thyroid Enlargement Graves' Disease): Patients with Grade II or III thyroid enlargement. | | | | | | | |
